# Design of the Building Research in CRC prevention (BRIDGE-CRC) trial: a 6-month, parallel group Mediterranean diet and weight loss randomized controlled lifestyle intervention targeting the bile acid-gut microbiome axis to reduce colorectal cancer risk among African American/Black adults with obesity

**DOI:** 10.1186/s13063-023-07115-4

**Published:** 2023-02-15

**Authors:** Andrew McLeod, Patricia Wolf, Robert S. Chapkin, Laurie A. Davidson, Ivan Ivanov, Michael Berbaum, Lauren R. Williams, H. Rex Gaskins, Jason Ridlon, Jen Sanchez-Flack, Lara Blumstein, Linda Schiffer, Alyshia Hamm, Kate Cares, Mirjana Antonic, Beatriz Penalver Bernabe, Marian Fitzgibbon, Lisa Tussing-Humphreys

**Affiliations:** 1 Institute for Health Research and Policy, University of Illinois Chicago (UIC), Chicago, IL USA; 2 Department of Nutrition Science, Purdue University, West Lafayette, IN USA; 3 Department of Nutrition, Program in Integrative Nutrition & Complex Diseases, and Center for Environmental Health Research, Texas A&M University, College Station, TX USA; 4 Department of Veterinary Physiology & Pharmacology, and Center for Environmental Health Research, Texas A&M University, College Station, TX USA; 5 Mile Square Health Center, University of Illinois Chicago, Chicago, IL USA; 6 Department of Animal Sciences, University of Illinois Urbana-Champaign, Urbana, IL USA; 7 Division of Nutritional Sciences, University of Illinois Urbana-Champaign, Urbana, IL USA; 8 Carl R. Woese Institute for Genomic Biology, University of Illinois Urbana-Champaign, Urbana, IL USA; 9 Cancer Center at Illinois, University of Illinois Urbana-Champaign, Urbana, IL USA; 10 Department of Biomedical and Translational Sciences, University of Illinois Urbana-Champaign, Urbana, IL USA; 11 Department of Pathobiology, University of Illinois Urbana-Champaign, Urbana, IL USA; 12 Department of Pediatrics, University of Illinois Chicago, Chicago, IL USA; 13 University of Illinois Cancer Center, University of Illinois Chicago, Chicago, IL USA; 14 Department of Kinesiology and Nutrition, University of Illinois Chicago, Chicago, IL USA; 15 Department of Biomedical Engineering, University of Illinois Chicago, Chicago, IL USA

**Keywords:** Cancer health disparities, Colorectal cancer, Mediterranean diet, Weight loss, Gut microbiome, Bile acids, Nutrition

## Abstract

**Background:**

Among all racial/ethnic groups, people who identify as African American/Blacks have the second highest colorectal cancer (CRC) incidence in the USA. This disparity may exist because African American/Blacks, compared to other racial/ethnic groups, have a higher prevalence of risk factors for CRC, including obesity, low fiber consumption, and higher intakes of fat and animal protein. One unexplored, underlying mechanism of this relationship is the bile acid-gut microbiome axis. High saturated fat, low fiber diets, and obesity lead to increases in tumor promoting secondary bile acids. Diets high in fiber, such as a Mediterranean diet, and intentional weight loss may reduce CRC risk by modulating the bile acid-gut microbiome axis. The purpose of this study is to test the impact of a Mediterranean diet alone, weight loss alone, or both, compared to typical diet controls on the bile acid-gut microbiome axis and CRC risk factors among African American/Blacks with obesity. Because weight loss or a Mediterranean diet alone can reduce CRC risk, we hypothesize that weight loss plus a Mediterranean diet will reduce CRC risk the most.

**Methods:**

This randomized controlled lifestyle intervention will randomize 192 African American/Blacks with obesity, aged 45–75 years to one of four arms: Mediterranean diet, weight loss, weight loss plus Mediterranean diet, or typical diet controls, for 6 months (48 per arm). Data will be collected at baseline, mid-study, and study end. Primary outcomes include total circulating and fecal bile acids, taurine-conjugated bile acids, and deoxycholic acid. Secondary outcomes include body weight, body composition, dietary change, physical activity, metabolic risk, circulating cytokines, gut microbial community structure and composition, fecal short-chain fatty acids, and expression levels of genes from exfoliated intestinal cells linked to carcinogenesis.

**Discussion:**

This study will be the first randomized controlled trial to examine the effects of a Mediterranean diet, weight loss, or both on bile acid metabolism, the gut microbiome, and intestinal epithelial genes associated with carcinogenesis. This approach to CRC risk reduction may be especially important among African American/Blacks given their higher risk factor profile and increased CRC incidence.

**Trial registration:**

ClinicalTrials.gov NCT04753359. Registered on 15 February 2021.

**Supplementary Information:**

The online version contains supplementary material available at 10.1186/s13063-023-07115-4.

## Administrative information

**Table:** 

**Funding**	National Cancer Institute (R01CA250390-01A1)
**Sponsor**	National Cancer Institute 9000 Rockville Pike Bethesda, MD 20892
**Role of Sponsor**	The study sponsor shall have no influence over the design, implementation, conduct, results or interpretations of the results of this trial.

The completed SPIRIT checklist is attached as Additional file 1.

## Introduction

Colorectal cancer (CRC) is the third most common cancer in the USA, with 151,030 new cases and 52,580 CRC-related deaths expected in 2022 [1]. Among all racial/ethnic groups, people who identify as African American/Blacks (AA/Bs) have the second highest CRC incidence and highest CRC mortality in the USA [1]. This disparity may exist in part because AA/Bs, compared to other racial/ethnic groups, have a higher prevalence of risk factors for CRC, such as higher body fatness [2, 3], lower dietary fiber consumption [3, 4], poorer diet quality [5, 6], and higher intake of saturated fats [7, 8] and animal proteins [3, 8, 9].

While there are several proposed mechanisms linking these modifiable risk factors with CRC, one potential mechanism is the bile acid (BA)-gut microbiome axis [10–12]. This is the two-way interaction between the gut microbiome and BAs that may explain the etiology of several diseases [13]. High levels of dietary fat and animal protein increase fecal and circulating BAs and promote a shift in amino acid conjugation of the BA pool from glycine to taurine [14, 15]. The gut bacteria *Bilophila wadsworthia* and *Clostridium scindens* can increase their abundance from, and metabolize taurine-conjugated BAs to increase fecal levels of the tumor-promoting/secondary bile acid deoxycholic acid (DCA) and to increase concentrations of hydrogen sulfide (H_2_S), a carcinogen that can trigger dysregulated cell-cycle progression and DNA repair responses [12]. Importantly, *B. wadsworthia* is significantly more abundant in uninvolved mucosa of CRC cases versus controls among AA/Bs but not non-Hispanic Whites [7]. Furthermore, H_2_S dampens production of butyrate [16], an anti-neoplastic microbial metabolite [17]. Hence, the gut microbiota may explain to some extent why higher intakes of fat and animal protein sometimes observed in AA/Bs compared to other race/ethnicities place them at increased risk of CRC.

The gut microbiota may also help explain how weight loss (WL), increased fiber, and decreased fat and animal protein consumption may decrease CRC risk in this subgroup. Aleman et al. demonstrated that a VLCD producing 10% WL reduced fecal BAs and DCA, and yielded a higher ratio of glycine-conjugated BAs, which correlated directly with gut microbial compositional changes [18]. Biemann et al. showed that a 5–15% WL from a lifestyle intervention significantly reduced circulating BA levels compared to a control group [19]. Lastly, in O’Keefe et al., increasing fiber consumption and decreasing saturated fat and animal protein in in AA/Bs for 2 weeks increased fecal butyrate and reduced *B. wadsworthia* abundance, fecal DCA levels, and colonic histological biomarkers of CRC [8].

Based on these prior studies, we hypothesize that the largely plant-based and fiber-rich Mediterranean diet (MedDiet), which is associated with lower circulating DCA [20], higher abundance of fecal bacteria capable of producing butyrate [21], and decreased CRC risk in observational studies [22], will have robust positive effects on the BA-gut microbiome axis among persons with elevated levels of colonic bacteria capable of producing sulfide, including AA/Bs. Furthermore, because weight loss and a healthful diet may have an additive effect on disease risk reduction [23], we hypothesize that WL via caloric restriction combined with a MedDiet will induce the greatest weight loss, the greatest improvements in the microbiome-BA axis, and the greatest reduction in intestinal gene expression associated with carcinogenesis.

To our knowledge, no studies have tested this hypothesis. To fill this gap, we will conduct a randomized controlled trial (RCT) enrolling AA/B, middle to older aged adults with obesity to examine the effects of a MedDiet alone (Med-A), WL through lifestyle intervention alone (WL-A), or a calorie-restricted MedDiet for WL (WL-Med) compared to typical diet controls (TDC), on BA metabolism, the gut microbiota, and intestinal epithelial markers associated with carcinogenesis.

## Methods

### Study design

**Fig. 1:**
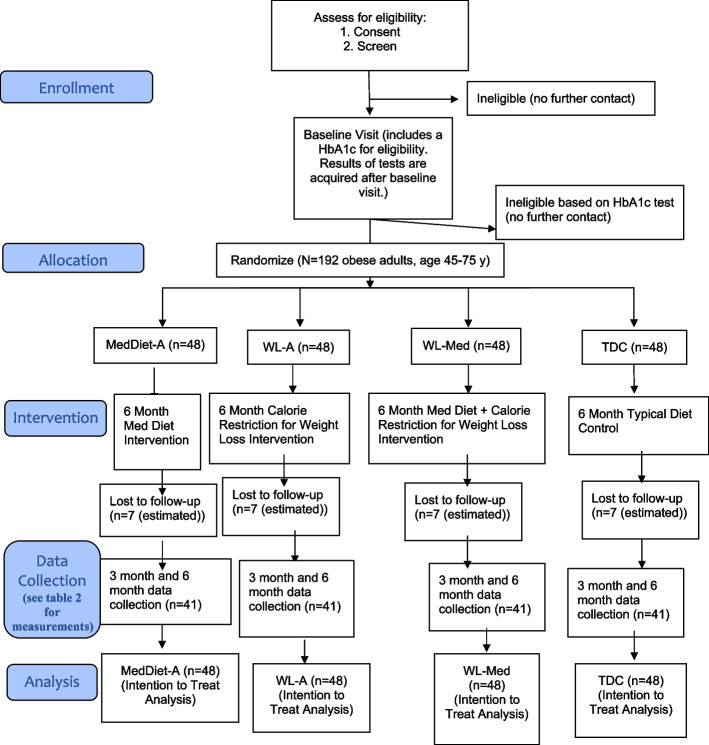
SPIRIT figure

The Building Research in CRC prevention (BRIDGE-CRC) trial is a parallel, four-arm, superiority RCT that will enroll 1922 adults aged 45–75 years old with obesity and who self-identify as AA/B. We will randomly assign 48 participants to each arm: Med-A, WL-A, WL-Med, and TDC. The interventions are 6 months in duration, with assessment of study outcomes occurring at baseline, month 3, and study end. See SPIRIT figure (Fig. 1) for participant flow.

### Recruitment

Participants will be identified through electronic medical records (EMR) of the University of Illinois Health and Hospital System (UI Health). They will also be recruited through email listservs, social media, presentations at senior centers, and flyers at UI Health and health fairs. We will aim to recruit 30% male and 70% female participants given the underrepresentation of males within dietary and weight loss interventions [24]. We will recruit participants in three separate, approximately equal cohorts.

### Inclusion criteria

Potentially eligible participants will be screened in two phases—the first, via telephone and the second, in-person. Inclusion criteria to be assessed over these two phases include age (45–75 years), self-identify as AA/B, body mass index (BMI) 30–50 kg/m^2^, willing and able to participate in all procedures, willing to be randomized to 1 of the 4 study arms, and willing to provide informed consent. Participants must also understand English, have access to a smartphone, be familiar with digital meeting platforms, and plan to reside in the Chicago area for the duration of the intervention. Doctor’s approval will be required for the following conditions: recent-onset chest pain, tightness, or pressure during physical activity; falling, feeling unsteady, or using an assistive device while standing or walking; other health concerns about starting an exercise program; or systolic blood pressure ≥ 160 mmHg or diastolic blood pressure ≥ 100 mmHg.

### Exclusion criteria

Exclusion criteria include the following: hypertensive crisis (defined as a blood pressure > 180/115mmHg with symptoms such as chest pain and headache); renal disease; autoimmune disorders; immunodeficiency; malabsorptive disorders; significant gastrointestinal and hepatic diseases; severe ischemic heart disease; unable to exercise due to emphysema; chronic bronchitis or asthma; bariatric surgery (excluding lap bands not filled with saline); regularly drinks 3–4 alcoholic drinks per day or more; illicit drug use (other than marijuana based on self-report); combustible tobacco use; uncontrolled diabetes based on hemoglobin A1c (HbA1c) > 9.0% (assessed after baseline data collection); eating disorder; any cancer treatment within the past 12 months; history of CRC; genetic predisposition to CRC (e.g., Lynch syndrome); difficulty walking; weight > 450 lbs. (weight limitation of the DXA scanner); currently adhering to a MedDiet based on the 21 point Mediterranean Eating Pattern for Americans (MEPA)-III screener [25] (adherence defined as a score ≥ 13); self-reported WL of 10 lbs. in the past 3 months; preparing for gastric bypass surgery or other bariatric surgery; unable or unwilling to adopt a MedDiet; oral or IV antibiotic use in the 3 months prior to data collection; takes prebiotics, synbiotics, dietary fiber supplements, or laxatives ≥ 3 times per week; night-shift work, unwilling to stop use of prebiotics, probiotics, synbiotics, dietary fiber supplements, or laxatives from 4 weeks prior to baseline data collection up until the last data collection period; pregnancy; or active COVID-19 infection within 5 weeks prior to data collection.

### Informed consent

We will obtain an IRB waiver of consent to identify potential research participants using the University of Illinois Health and Hospital System EMR. Written, informed consent (see Additional file 2 for consent form) and HIPAA authorization will be obtained in-person or via video conferencing by research staff trained and certified to obtain informed consent. The written consent will be reviewed in detail and used as a guide for the person obtaining the consent. Participants will be asked if they consent or not to having their biospecimens archived for use in future, ancillary studies. Participants are encouraged to ask questions throughout the process. The informed consent procedure generally takes 15–30 min to complete. Participants are given a signed copy of the consent to bring home and informed again that they are free to withdraw at any time. Participants will be paid an honorarium for their participation in the full study. $70 will be provided for baseline data collection, $70 for month 3, and $100 for final assessment plus $240 either in food delivery service or as a financial payment for the TDC participants.

### Randomization

The data manager, who has no contact with participants, will use the REDCap randomization module to randomly assign participants to (1) Med-A, (2) WL-A, (3) WL-Med, or (4) TDC, in a 1:1:1:1 allocation ratio. Stratified block randomization will be adopted to ensure good balance among the four study groups with respect to the selected covariates: sex, age (45–60 or 61–75 years), and baseline BMI (30–39.99 or 40–50 kg/m^2^). The randomization allocation will be created in SAS and imported into the REDCap randomization module.

### Dissemination policy

Results will be made public via timely publications in the best journal possible and at academic conferences. Authorship will be defined per authorship definitions provided by the International Committee of Medical Journal Editors [26].

## Interventions

### Behavior change models

The interventions incorporate constructs of the Social Cognitive Theory [27] and Self-Determination Theory [28] that include self-efficacy, social support, motivation, and barrier reduction. Med-A, WL-A, and WL-Med use dietary monitoring, individualized goal setting, and problem solving to increase mastery and goal achievement in small incremental steps to enhance self-regulatory skills. For the MedDiet interventions (Med-A, WL-Med), learning to prepare MedDiet-friendly foods is a major aspect of dietary pattern adoption mastery and goal achievement. The intervention is implemented in 24 weekly one-on-one sessions and through a private Facebook group containing videos and posts. If a participant does not want to participate in the social media group, the social media content is sent via email. An overview of the first 5 weeks of the intervention by study arm is included in Additional file 3: Table S1, Additional file 4: Table S2, and Additional file 5: Table S3.

### Intervention instructors

The intervention instructors have experience conducting nutrition interventions and community-based nutrition education, with the lead interventionist having registered dietitian credentials. There is a separate interventionist for each study arm.

### Individual sessions

Participants in Med-A, WL-A, and WL-Med will meet with their interventionist once weekly in-person or via Zoom (based on participant preference) for 24 sessions. Two of these sessions will be “floating” to allow for holidays that may interfere with the once-weekly plan. Weekly session content is manualized and includes time to address successes and barriers and problem solving using motivational interviewing techniques. Week one is planned as a 1-h session, with the remaining 23 weeks planned as 30-min sessions. For participants randomized to Med-A and WL-Med, the interventionist will instruct on and support the adoption of an eating pattern consistent with a MedDiet using an individualized MedDiet exchange list and companion guide [29, 30]. The exchange list will contain several categories of foods and beverages characteristic of a MedDiet (e.g., low-fat dairy). Participants will be advised to consume a specific quantity of food and beverage from each category, but will be free to choose which foods and beverages from each category they would like to consume.

### Social media private groups

The WL-A, Med-A, and WL-Med interventionists will each lead their own private Facebook group. Each week, the interventionist will host a Facebook Live event providing an overview of the topics and content to be discussed in individual sessions and posted to the social media page. The live event will also be an opportunity for participants to ask questions. The Facebook Live content will be posted for participants who are unable to join in real-time. Weekly content will also include instructional videos (e.g., how to count MedDiet exchanges), existing web content pertinent to the study arm (e.g., YouTube cooking videos), and cooking demonstrations created by the study team and infographics. The number of weekly postings will be equivalent across the study arms. Participation at live events and views and completion analytics are available at the individual level and will be used to evaluate interaction with the social media content. Those opting out of the social media group will be asked to confirm receipt of the weekly content via email.

### Intervention calorie and nutrient recommendations

The Med-A and WL-Med arms will receive similar recommendations regarding macro-and micronutrient composition, with an emphasis on consuming fruits, vegetables, whole grains, plant proteins such as nuts and legumes, olive oil as the main dietary fat, and red wine. Fast foods, red and processed meats, fried foods, pastries, refined carbohydrates (e.g., white bread and white rice), sugary beverages, and high-fat dairy products will be discouraged. Recommended daily exchanges will be based on individual caloric needs to maintain weight (Med-A) or to achieve approximately 5% WL from baseline at study end (WL-Med). For participants randomized to WL-A, the focus will be on daily calorie restriction (−500 to −750 kcal/day) to achieve approximately 5% WL at study end. The content for the WL-A arm will be adapted from the Diabetes Prevention Program open-source intervention materials [31]. Energy needs for the WL-Med and WL-A arms will be determined using the Mifflin St. Jeor equation with an activity factor based on baseline physical activity level [32]. Balancing alcohol’s effect on BA metabolism while respecting participants’ dietary preferences, interventionists will recommend that alcohol comprise at most 1 drink per day for females and males and when possible consumed with meals. See Table 1 for more details regarding the intervention diet recommendations.

**Table 1:** Intervention calorie and nutrient recommendations

Dietary component	Med-A	WL-Med	WL-A
**Calories**	No caloric deficit	500–750 kcal/day deficit	500–750 kcal/day deficit
**Total fat**	~35% of total calories	~35% of total calories	~ 30% of total calories
***Saturated fat***	~5% of total calories	~5% of total calories	< 10% of total calories
***Monounsaturated fat***	~20% of total calories	~20% of total calories	No specific recommendation
***Polyunsaturated fat***	~10% of total calories	~10% of total calories	No specific recommendation
**Carbohydrates**	~40% of total calories	~40% of total calories	No specific recommendation
**Protein**	~20% of total calories	~20% of total calories	No specific recommendation
**Alcohol**	≤1 drink per day females and males (if alcohol is usually consumed)	≤1 drink per day females and males (if alcohol is usually consumed)	No specific recommendation
**Sodium**	Limit sodium	Limit sodium	Limit sodium
**Fiber**	25–35 g/day	25–35 g/day	No specific recommendation

### Food delivery

The Med-WL and Med-A groups will receive food boxes with ingredients to make the recipe for the dish prepared in the cooking skills video content for the corresponding week. The food will be sourced and delivered by Top Box Foods (Chicago, IL), a community-based non-profit with a mission to increase access to affordable and healthy foods. The deliveries will begin during week 2 of the intervention and continue on a biweekly basis until week 14, at which point deliveries will occur every fourth week, with a total of 9 deliveries throughout the 24-week study. Additionally, participants will be provided with a pantry box in the first session which will include shelf stable items to be used in recipes for later sessions.

### Physical activity

The Med-A and control groups will be asked to maintain their baseline physical activity pattern, and the WL-A and WL-Med groups will be asked to progress toward achieving 10,000 steps daily. A fitness instructor will engage with participants through the FitBit platform to monitor step counts and to encourage and motivate participants to increase daily steps. The fitness instructor will also create and post social media content (described more below) to provide strategies and motivation around increasing daily steps. The WL-A and WL-Med interventionists will also post challenges to the private Facebook groups to encourage increasing daily steps among their participants.

### Control (TDC) participants

A research team member will meet individually with the TDC participants at the start of the intervention. They will not receive information about making diet or physical activity changes and will be asked to maintain current diet and physical activity behaviors. Additionally, they will receive weekly health newsletters about non-diet related health topics (e.g., flu prevention). All WL-Med materials will be offered to the TDC participants in a self-guided format following the intervention.

### Adherence

Several methods will be used to assess intervention adherence. First, weekly attendance at individual sessions will be tracked. Participants missing two consecutive individual sessions will be called and text messaged by the interventionist to encourage attendance. Second, engagement with weekly social media content will be monitored using Facebook analytics which is reported on the individual level. Participants not engaging with the social media content for two consecutive weeks will be reminded to engage with the content. Participants receiving social media content via email who fail to confirm receipt for two consecutive weeks will be contacted and asked to confirm receipt of the materials. Third, body weight will be monitored weekly in the WL-A and WL-Med groups. Participants will be asked to weigh in person or transmit a photo via secure text message during their weekly one-on-one meetings. Participants who lose < 1 lb. per week for 3 consecutive weeks will discuss with their interventionist barriers to adopting a calorie-restricted eating plan and problem solve accordingly. If necessary, the interventionist will revise the calorie prescription (WL-A and WL-Med) and the number of daily MedDiet exchanges (WL-Med). For the Med-A group, we will record the weight of participants monthly to confirm adherence. Lastly, adherence to the MedDiet will be monitored during the first 6 weeks of the intervention among the Med-A and WL-Med participants. During weekly meetings with the interventionist, participants will complete the MEPA-III screener [25]. Those unable to increase adherence to a Med Diet will discuss with their interventionist barriers to adopting a MedDiet and problem solve accordingly.

### Intervention fidelity

To assure fidelity, intervention sessions will be semi-structured in a manualized format. Given the individualized nature of the sessions, the interventions cannot be fully scripted. A semi-structured approach will allow for use of a fidelity checklist itemizing the expected general content for sessions. The interventionists will be observed by senior staff once weekly, and the fidelity checklist will be completed. Based on the fidelity checklist, senior staff will meet with the interventionists to provide feedback and to review progress, concerns, and address deviations from the intended intervention content. This approach has worked well in our previous trial [33].

## Study outcomes and data collection procedures

### Outcomes

The primary study outcomes are total circulating and fecal BAs, taurine-conjugated BAs, and DCA. Secondary outcomes include body weight, body composition, dietary change (i.e., MedDiet adherence), physical activity (i.e., steps per day), metabolic risk, circulating cytokines (e.g., IL-6, TNFα), gut microbial community structure and composition, fecal short chain fatty acids (SCFAs) (i.e., butyrate, acetate, propionate), and exfoliated intestinal cell gene expression levels. Data collection will take place at UIC and by telephone at baseline, month 3, and study end. All data collection staff will be trained and certified in informed consent, testing procedures, specimen processing, and data management. All procedures will be written and stored electronically. Major changes to study design will first be reviewed and approved by the IRB before implementation. The primary data collectors and lab technicians will be blinded to randomization group assignment by having the assignment hidden from view in their individual REDCap account.

### Participant confidentiality

The confidentiality of participants will be safeguarded using unique participant identification numbers/codes. Participant biospecimens will be kept in a locked lab until analysis. The biospecimens will be labeled with ID, date, and specimen type only. All survey data will be directly entered into the Research Electronic Data Capture (REDCap) data management system using data structure developed by the study team [34]. Only the investigators and members of the research team will have access to participant data files, biospecimens, and REDCap data structure. Datasets used for analysis will not contain any direct identifiers.

### Measurements

See Table 2 for an outline of measurements taken at each data collection time point.

**Table 2:** BRIDGE-CRC trial measurements

Data type	Baseline	Month 3	Month 6
*Anthropometrics*			
Height	X		
Weight^a^	X	X	X
Body composition (DXA)	X		X
*Inflammatory markers*			
Systemic inflammation (hs-CRP)	X	X	X
Circulating cytokines (GM-CSF, IL-2, IL-4, IL-6, IL-8, IL-10, TNFα, and IFNγ)	X	X	X
*Metabolic risk*			
Glucose, mg/dL	X	X	X
Insulin, uIU/mL	X	X	X
Hemoglobin A1c, %	X	X	X
HOMA-IR (calculated from glucose and insulin)	X	X	X
Blood pressure, mmHg	X		X
*Dietary intake*			
VioScreen food frequency questionnaire (FFQ)	X		X
24-h diet recall (two per timepoint) (ASA24)	X	X	X
MEPA-III MedDiet screener	X	X	X
Medication use	X	X	X
Physical activity (steps per day, FitBit)	X		X
Microbiota composition (16S rRNA)	X	X	X
Predicted gene abundance, metabolic pathways, and microbial network analysis	X	X	X
Quantification of the following genes via PCR: *dissimilatory sulfite reductase A* (pan*dsrA*), *B. wadsworthia*-specific *dsrA*, *16S rRNA*, *Desulfovibrio* spp.-specific *16S rRNA*, BA *7α-dehydratase* from *C. scindens*, *butyryl-CoA transferase* gene (*BcoA*)	X	X	X
Fecal short chain fatty acids (uM) (e.g., butyrate, acetate, and propionate)	X	X	X
Fecal and circulating bile acids (uM) (i.e., total BAs, total unconjugated BAs, taurine- and glycine-conjugated BAs, and CA, CDCA, LCA, and DCA)	X	X	X
Exfoliated intestinal cell gene expression analysis	X	X	X
Surveys			
Demographics	X		
Health history, including bowel health	X		X
Sleep quality	X		X
Food away from home	X		X
*Psychosocial variables*			
Discrimination	X		
Food security	X		X
Depressive symptoms	X		X
Stress	X		X
Recent trauma	X		X
Anxiety	X		X

*Abbreviations: BA* bile acid, *CA* cholic acid, *CDCA* chenodeoxycholic acid, *DCA* deoxycholic acid, *DXA* dual energy X-ray absorptiometry, *GM-CSF* granulocyte-macrophage colony-stimulating factor, *HOMA-IR* Homeostatic Model Assessment for Insulin Resistance, *hs-CRP* high-sensitivity C-reactive protein, *IFN* interferon, *IL* interleukin, *LCA* lithocholic acid, *MedDiet* Mediterranean diet, *MEPA* Mediterranean Eating Pattern for Americans, *TNF* tumor necrosis factor

^a^Weight will also be recorded as needed throughout the intervention to monitor compliance

### Anthropometrics

Height will be measured, in duplicate, to the nearest 0.1 cm at the beginning of the study using a fixed stadiometer (Seca, Birmingham, UK). Weight will be measured, in duplicate, to the nearest 0.1 kg using a digital scale (Tanita, Arlington Heights, IL, USA). Body composition will be measured with dual energy X-ray absorptiometry (DXA) on a General Electric Lunar iDXA machine (GE Healthcare, Chicago, IL, USA).

### Blood-based biomarkers

Participants, who have fasted for at least 12 h, will undergo a blood draw, and we will retain whole blood and derive serum, plasma, and buffy coat using centrifugation at 3000 RPM for 15 min at 4°C. Blood products will be sent to Quest Diagnostics (Wood Dale, IL, USA) immediately or stored in a −80°C freezer for later analysis.

### Inflammatory markers

Systemic inflammation will be operationalized with CRP and measured with an immunoturbidimetric assay at Quest Diagnostics (Wood Dale, IL, USA). Circulating cytokines (GM-CSF, IL-2, IL-4, IL-6, IL-8, IL-10, TNFα, and IFNγ) will be measured with the Bio-Plex human cytokine assay (Bio-Rad, Hercules, CA, USA) at the UIC Research Resources Center (Chicago, IL, USA).

### Metabolic risk

Glucose, insulin, and HbA1c will be measured by Quest Diagnostics (Wood Dale, IL, USA) using spectrophotometry, an immunoassay, and an enzymatic assay, respectively. Homeostatic model assessment of insulin resistance (HOMA-IR) will be calculated according to a published formula: (fasting glucose [mmol/l]×fasting insulin [μU/ml])/22.5 [35].

### Blood pressure

Blood pressure will be measured with an electronic, automated blood pressure machine (Omron 3 Series, Kyoto, Japan). Participants will rest for a minimum of 5 min before having their blood pressure measured twice in a seated position. Participants will be instructed to rest for 1 min between the two blood pressure readings.

### Dietary intake

Habitual diet will be assessed with the validated, online, VioScreen food frequency questionnaire (FFQ) [36]. Recent dietary habits will be collected and analyzed using the Automated Self-Administered 24-hour (ASA24) Dietary Assessment Tool, version (2020), developed by the National Cancer Institute, Bethesda, MD, USA [37]. The ASA24 will be completed twice at each data collection point. While both instruments are designed to be self-administered, they will instead be administered by trained and certified research staff to reduce recall bias. A MedDiet adherence score first created by Panagiotakos et al. [38] and later adapted to an American population by Tangney et al. [39] will be calculated from both the FFQ and ASA24 data. Lastly, the MEPA-III MedDiet screener [25], developed by Tangney et al., will be used not only as a screener but to quickly assess MedDiet adherence at each data collection timepoint for the Med-A and WL-Med arms. It consists of 21 items divided into 9 categories (e.g., fruits, vegetables, nuts and seeds) and assesses frequency and quantity of intake of foods encouraged and discouraged by a MedDiet eating pattern.

### Medication use

We will catalog prescription medications, over the counter drugs, and dietary supplements taken in the past 30 days.

### Physical activity

Participants will wear a fitness tracker (FitBit, San Francisco, CA, USA) for 7 days. We will access steps, heart rate, and sleep data from the FitBit with a third-party application, FitaBase (San Diego, CA, USA).

### Stool collection

Stool will be collected by participants 0–48 h prior to each of the three data collection visits using a kit provided by study staff. They will be instructed to store collected stool in a commercial refrigerator and to transport the stool on an ice pack to each visit. The stool will be stored in a refrigerator during the study visit. After the visit, it will be processed immediately for exfoliomics analysis (see below), with the remaining stool being aliquoted, flash frozen in liquid nitrogen, and then stored at −80°C until further analysis.

### Fecal microbiota composition

Microbial DNA will be extracted with the Qiagen DNeasy PowerLyzer Powersoil Kit (Qiagen, Hilden, Germany) as done previously [7]. The UIC Genomics core will polymerase chain reaction (PCR) amplify genomic DNA with primers CS1_515F and CS2_806R (modified from the set used by the Earth Microbiome Project) targeting the V4 region of microbial small subunit ribosomal RNA (rRNA) genes. Amplicons will be generated using a two-stage PCR protocol, described previously [40, 41]. The V4 region of the 16S rRNA gene will be sequenced with the Illumina MiSeq platform to generate 2×250 bp paired end reads per sample [42]. Environmental controls will be included in the sequences to distinguish from any contaminants in reagents or the lab environment.

### Fecal microbiome analysis

16S rRNA reads will be processed with DADA2 [43]. After quality filtering and removing low abundance (<0.5%) reads, Silva v132 reference sequence database [44] will be used for taxonomical assignment of the Amplicon Sequence Variants (ASVs).

### Predicted gene abundance, metabolic pathways, and microbial network analysis

Employing the software PiCRUSt2 [45], we will predict gene abundance and most probable metabolic pathways using ASVs as input. The software SPIEC-EASI will be used to model microbial network configurations [46].

### Quantification of microbial gene targets

Quantitative PCR (qPCR) will be performed with a 7500Fast Real-Time PCR system (Life Technologies, Carlsbad, CA, USA) and validated primers, as described previously [7, 8, 47], using microbial DNA isolated as explained above. Functional gene targets for sulfide production will be measured including degenerate dissimilatory sulfite reductase A (pan-dsrA) and *B. wadsworthia*-specific dsrA (dsrA-Bw). Primers targeting 16S rRNA genes will be used to measure total bacterial abundance as well as *Desulfovibrio* spp. The BA 7α-dehydratase (*baiE*) gene from DCA producing *C. scindens*, and *butyryl-CoA transferase* gene (*BcoA*) will also be quantified. An internal standard will be used.

### Fecal SCFAs

Flash frozen stool will be transferred into plastic tubes; 2,2-dimethylbutyric acid will be added at 1 mmol/L as internal standard, vortexed and centrifuged, and supernatant filtered through a Millex-GS filter (Millipore, Burlington, MA, USA) and refiltered with a microconcentrator (Millipore, Burlington, MA, USA). The final filtrate will be analyzed using an Agilent Technologies 6890N Network GC System (Santa Clara, CA, USA) with a flame ionization detector for SCFAs. A mixed-SCFA standard solution will be prepared using high purity (0.99%) reagents (Sigma, St. Louis, MO, USA). Fecal SCFA concentrations will be computed by using a peak area ratio of the sample profile relative to the internal standard. Analysis will be conducted by the UIC Mass Spectrometry, Metabolomics and Proteomics Facility.

### Fecal and circulating bile acids

All major primary (e.g., CA and CDCA) and secondary fecal and circulating BAs (e.g., DCA and LCA) as well as their glycine and taurine conjugates will be assessed. Total BAs and total unconjugated BAs will also be measured. Samples will be extracted, and supernatants will be dried and resuspended for liquid chromatography/mass spectroscopy analysis following validated and published methods [48]. Authentic reference BAs will be purchased from Sigma-Aldrich (St. Louis, MO, USA) and Steraloids (Newport, RI, USA). Blind duplicate samples will be used to assess inter- and intra-batch variability. An internal standard will be used.

### Stool-based exfoliated intestinal epithelial cell analysis—exfoliomics

From stool preserved in Ambion Denaturation Solution, eukaryotic polyA+ RNA will be isolated using the Active Motif mTRAP Maxi kit followed by DNA removal with DNAFree (Invitrogen, Waltham, MA, USA) as previously described [49]. Illumina libraries will be created with 100 ng RNA using the Lexogen QuantSeq 3′ mRNA-Seq Library Prep Kit for Illumina (FWD) (Lexogen, Vienna, Austria) and unique molecular identifiers. The libraries will be generated following the adjusted protocol from the manufacturer for low input RNA and quantified using the Qubit 1x dsDNA HS assay kit (ThermoFisher Scientific, Waltham, MA, USA) and a Bioanalyzer High Sensitivity DNA Analysis kit (Agilent, Santa Clara, CA) following manufacturer’s instructions. Sequencing will be performed on an Illumina NovaSeq 6000 platform using standard Illumina protocols. RNA reads will be mapped with the Bowtie2 [50] using optimized parameters to the Ensembl GRCh38 human reference. Reads will be examined for quality control using FastQC and quantified using HTSeq-count [51, 52]. Sequencing reads will be filtered to remove any gene with less than 2 reads in more than 33% of the samples [49].

### Surveys

All non-dietary surveys will be electronic and self-administered. The measures include demographics (e.g., sex, household income), health history including bowel health (Bowel Health Questionnaire from NHANES [53]), sleep quality (Pittsburgh Sleep Quality Index [54]), discrimination (Everyday Discrimination Scale [55]), food security (US Household Food Security Survey Module [56]), depressive symptoms (Patient Health Questionnaire-8 (PHQ-8) [57]), perceived stress (Perceived Stress Scale (PSS-10) [58]), recent trauma (Recent Traumatic Events Scale [59]), anxiety (Generalized Anxiety Disorder scale, GAD-7 [60]), and food (eaten) away from home [61].

## Statistical analysis plan

### Sample size

We calculated the sample size to detect group differences in three circulating and fecal BA outcomes (total BAs, taurine-conjugated BAs, and DCA) using results reported by Biemann et al. [19] and Aleman et al. [18] as a guide. In Biemann et al. [19], data were summarized as medians, interquartile ranges, and sample sizes; we estimated the corresponding means and standard deviations [62]. Estimated effect sizes for diet intervention on serum total BAs and taurine-conjugated BAs are 0.75 and 1.2 (assuming the correlation coefficient between pre and post measurements is 0.9 as commonly observed in repeated assays). Estimated effect size on fecal DCA is 0.67 using the raw data provided by Aleman (assuming change in the control group is 0) [18, 62]. For each dependent variable, the overall level of type I error is set at *α* = 0.05; that is, no adjustment is made for correlation among dependent variables and hence tests of these variables are not independent. The sets of variables examined in Hypotheses 1 and 2 should be interpreted as related. With 4 groups, there are 6 pairwise contrasts among cell means at a given occasion; we plan to test 3 of these using *t*-tests at the Bonferroni-adjusted *α*^′^ = 0.05/3 ≈ 0.017 significance level. For the smallest effect size 0.67, we need 41 subjects per group to afford power of 0.80 at *α*^′^ = 0.017. Note that in all cases these are one-sided tests of superiority in that change in one group’s mean is hypothesized to be greater than change in that of another group [63, 64]. For the smallest effect size, we need 164 subjects to complete the study, which offers ample power for the other effect sizes. Assuming 15% dropout at 6 months based on our previous studies, the recruitment target is 192 subjects (48 per arm).

Normal continuous variables will be presented as mean ± standard deviation. Continuous variables which cannot be transformed to achieve normality will be presented as median (interquartile range). Discrete variables will be presented as percentages.

Statistical analysis will be conducted using SAS v9.4 (Cary, NC). Independent and dependent *t*-tests will be employed to investigate differences in outcome measures between and within study groups, respectively. Any non-normal variables which cannot be transformed will be analyzed with the Mann-Whitney *U* and Wilcoxon signed-rank tests. The effect of the interventions on change in primary outcomes at mid- and post-intervention will be examined using repeated-measures linear models with restricted maximum likelihood estimation and a fully specified covariance matrix in SAS [65–67]. Such modeling can handle missing data as long as the data is missing at random, making this method ideal for our intention-to-treat analysis. Baseline covariates including the stratification variables for randomization will be adjusted for in all models. We will determine Spearman’s rank correlations between changes in circulating and fecal BAs and changes in fasting glucose, insulin, HOMA-IR, and circulating pro-inflammatory cytokines. We will also determine partial correlations for the above by adjusting for pertinent covariates, such as BMI.

### Statistical analysis of the 16S rRNA data and predicted microbial metabolic pathways

ASV counts will be normalized using cumulative sum scaling [68]. Differences in community richness, evenness, and structure [alpha (i.e., Shannon index) and beta diversity (i.e., Unifrac [69] and Bray-Curtis distances)] between groups will be determined using PERMANOVA from vegan R package. Generalized linear mixed models (GLMMs) will determine the phenotypic variables, cardiometabolic outcomes, and BA-associated variables associated with each microbial ASV and genus level as implemented in MasLin [70], with treatment group as the main fixed factor, adjusting for important covariates, such as age and medication usage. Multiple comparisons will be adjusted using false discovery rate (FDR) [71]. An FDR-corrected *p*-value ≤ 0.001 will be deemed significant. All analyses will be done with R using functions encoded in the phyloseq package [72]. ASVs will be employed to determine the most likely associated microbial genes that encode for enzymes and associated microbial metabolic pathways using PiCRUSt2 [45]. GLMMs will be employed to determine the genes and metabolic pathways associated with each intervention arm with MasLin [70]. Finally, we will determine the likelihood that the ensemble of microbial genes associated with the production and consumption of DCA and H_2_S are affected by the interventions.

### Statistical analysis of specific microbial target genes from qPCR and SCFAs

Microbial gene targets obtained via qPCR will be examined for distribution and transformed if appropriate to examine within and between group changes and differences at mid-study and post-intervention [7]. We will also examine associations between the microbial gene targets and serum and fecal BAs, circulating pro-inflammatory cytokines, and metabolic markers (e.g., fasting glucose) using principal component analyses, and canonical correspondence analyses as we have done previously [73]. Multiple linear regression analysis will be used to identify which determinant variable changes (e.g., fecal BAs) are independently associated with changes in microbial gene targets from baseline to mid-study and post-intervention, controlling for participant-level covariates such as age and medication usage.

### Gene expression analysis in exfoliated intestinal epithelial cells

We will test for differential gene expression (DE) in the exfoliome of participants using the generalized linear models available in edgeR, followed by FDR correction [71]. The DE genes will be interrogated by the Ingenuity Pathway Analysis (Qiagen, Hilden, Germany) software and enriched regulatory pathways will be identified as previously described [49].

### Feature selection based on linear discriminant analysis (LDA)

We will consider the regulatory genes expressed in the host exfoliome [74], the gut microbial ASVs, fecal BAs, and SCFAs as four categories of features in a classification problem that aims to detect sets of features (from the four feature categories) that best discriminate among the four groups. We will search all subsets of five or fewer features and test for significance using LDA [75, 76]. Sets with a low bolstered resubstitution misclassification error [77] (≤ 0.10) will be reported.

### Modeling of regulatory networks between the gut microbiome and human colonocytes

Our objective is to computationally model the host regulatory networks following exposure to the lifestyle interventions. Mathematical modeling of relationships in a gene regulatory network was originally proposed by Dougherty et al. [75] and further developed by members of our team [78]. We will use the concept of coefficient of determination (CoD) [79], which has previously been used to measure multivariate nonlinear gene interaction [75], and define the expectation that a master regulatory gene, when active, will be responsible for regulating one or more cascades of subsequent changes in the activity of other members of the respective pathway(s). This approach models a probabilistic relationship between regulators and regulated and derives the CoD of the master regulatory gene relative to the regulated [80]. The validation of the model for the case of gene regulatory networks has been performed as previously described [80–82] and can be readily extended to any regulatory cascade including bacterial species and microbially-produced metabolites (e.g., SCFAs, DCA) that interact with the host gut. Comparisons between the groups of participants will allow us to model the microbial consortia regulatory actions as modifiers of signaling cascades in colonocytes. Because the CoD does not imply directionality, we will incorporate existing knowledge of regulatory networks to constrain the space of possible solutions. We will also use this approach to discover novel regulatory relationships between the features used in our model.

### sCCA data integration

To assess the interdependencies among fecal microbiome, BAs, SCFAs, and host intestinal physiology in the intervention arms, we will deploy sparse Canonical Correlation Analysis (sCCA) [74, 83]. The groups of host genes, microbial ASVs, and microbial metabolites (i.e., SCFAs, DCA) identified by the 3-way sCCA will indicate potential biomarker sets for the participants in each one of the three intervention arms. We will also compare the 3-way correlative structures detected in each one of those groups to the others to elucidate the effects of the dietary interventions on the interactions between the host microbiota and the human gut relevant to CRC prevention and progression.

## Oversight and monitoring

### Organizational structure and responsibilities

This study will be conducted with a pre-specified organization structure assuming various responsibilities. Table 3 below details staff roles and duties.

**Table 3:** Organizational roles and responsibilities

Role	Responsibilities
Principal Investigators	- Oversee all research-related activity and personnel - Publish results in a timely manner
Data and Safety Monitoring Committee (DSMC)	-Review the study protocol for any major concerns prior to implementation -Regularly review and evaluate the accumulated study data for participant safety, study conduct, and progress -Make recommendations concerning the continuation, modification, or termination of the trial
Data Manager	-Organizes electronic data -Data verification -Assist with data safety
Project Director	-Manages recruitment and data collection -Manages budget and staff -Trains and certifies staff -Interfaces with IRB to maintain ethical standards

### Data and safety monitoring

The following information will be included in the yearly reports to the Chair of our data and safety monitoring committee: study event updates (e.g., changes in personnel, recruitment strategies, testing procedures), study enrollment (actual vs. expected), baseline data on participants, recruitment and retention flow diagram, participant retention data (loss to follow up, withdrawals), and adverse health event monitoring. An adverse health event is any untoward medical occurrence experienced by a participant during participation in the clinical study. These events can be non-serious or serious. A serious adverse event is any adverse event that results in one or more of the following outcomes:

- A life-threatening event
- Inpatient hospitalization or prolongation of existing hospitalization
- A persistent or significant disability/incapacity
- An important medical event based upon appropriate medical judgment
- Death

### Data and safety monitoring committee

The DSMC will consist of 12 independent experts reflecting the disciplines and medical specialties (e.g., clinical trials, biostatistics, nutrition, clinical medicine) necessary to interpret data from the clinical trial and to fully evaluate participant safety. They are independent of the study sponsor and have no competing interests. The primary responsibilities of the DSMC are outlined in Table 3. The PIs will meet with the DSMC annually and will present a written and verbal report. Additional meetings will be scheduled if warranted by the data or adverse events.

### Adverse event monitoring and reporting

The DSMC Chair will be notified of all study-related serious adverse events within 48 h of discovery. The Chair will decide if further action beyond what the research team has proposed is warranted. Additionally, participants will be asked to report each adverse health event in person at study visits or during the intervention sessions, or via telephone/email/text message. The principal investigators will consult and determine the severity and appropriate response or action to the reported adverse health event. All serious unanticipated problems or serious adverse health events will be reported to the UIC Institutional Review Board and Office for the Protection of Research Subjects.

UIC Office for the Protection of Research Subjects requires that all other problems (non-serious adverse events) be reported within 5 days of discovery and provide sufficient information to gauge severity and to complete reports. UIC Office for the Protection of Research Subjects has procedures in place to review the reports, and the PIs are notified if further review, changes to research protocol or consent form, or other action is required. The PIs will notify NIH should UIC IRB require further action after any unanticipated problem/adverse event is reported.

UIC has not set aside money to pay participants if they get ill or injured from being in the study. The only exception to this policy is if it is proven that a participant’s injury or illness is directly caused by the negligence of UIC.

## Discussion

The study described in this protocol will address potential solutions to reduce the high risk of CRC among AA/B adults by testing the ability of a MedDiet, weight loss, or both to reduce biological risk markers of CRC. This study will also address a potential mechanistic underpinning, the gut microbiome-bile acid axis, leading the way to more precise interventions.

To achieve these objectives, our study will use innovative and rigorous methods. First, measuring the exfoliome via excreted stool is less invasive than endoscopic sampling of the gut epithelium. It is also responsive to dietary interventions, can be used to measure the human gut transcriptome, and consists of epithelial cells which have been shown to become dysplastic even before stem cells do [49, 81, 84–88]. Here, we will further establish exfoliated intestinal epithelial cells in stool as non-invasive probes in human dietary lifestyle interventions. Second, we will employ statistical and computational analyses to examine the crosstalk between diet, weight loss, gut microbiome, and host intestinal physiology [89, 90]. Third, our study design allows us to determine the independent contributions of weight loss and dietary change to reducing biomarkers of CRC risk. Nearly all studies examining the effect of weight loss on CRC risk not only restrict calories or increase physical activity but also change the diets of participants [18, 19, 91–99]. Given the strong relationship between diet and CRC [3] as well as diet and the gut microbiota [100], any change in diet may mask or alter the independent effect of weight loss on cancer risk and gut microbiota. In our study, the weight loss arm is taught to lose weight only via caloric restriction and increased physical activity, not through dietary pattern change.

Our study is not without potential pitfalls and alternative outcomes. We have prepared for some of these eventualities. For example, participants who are not randomized to their preferred arm may pursue weight loss or dietary change independent of the intervention. If, for instance, a Med-A participant loses 5% of their body weight, this may obscure differences in outcomes between Med-A and any other arm. To help avoid this, we have implemented adherence measures to be taken throughout the study so that we may intervene when appropriate. We will also perform a per-protocol analysis, including only those who are adherent to their intervention arm. Similarly, given antibiotics’ profound impact on the gut microbiota, we will perform a sensitivity analysis whereby those who consume antibiotics during the intervention will be excluded from analysis to determine if results change significantly. Lastly, delivering the intervention remotely and by social media/email may dissuade less tech savvy participants during recruitment or disengage them after randomization. We determined this was a necessity to improve recruitment, as many would be participants were concerned with in-person classes given the continuing COVID-19 pandemic. Also, many work and were unable to attend in-person, group classes during working hours. Besides, remote intervention delivery is more convenient than in-person classes which would have required substantial travel time for participants, thus potentially improving retention. If successful in a remote format, our intervention could improve recruitment and retention for future studies.

## Conclusion

Our study has the potential to show that diet and/or weight loss can lead to reductions in biomarkers of CRC risk among a group of individuals under-represented in dietary and weight loss interventions but who bear a disproportionate burden of CRC incidence and mortality. It may also reveal potential mechanisms driving the effect of diet and weight loss, allowing for more precise and effectual interventions in the future. Ultimately, our findings could have a profound public health impact on reducing CRC risk among AA/Bs and translate into timely dissemination opportunities.

### Trial status

Recruitment began in March of 2022. Recruitment is expected to be complete by December 2024. The current Protocol version is 13, dated 9/26/22.

## Supplementary Information

**Additional file 1.** SPIRIT checklist.

**Additional file 2.** Consent form.

**Additional file 3: Table S1.** Med-A: Summary of the First 5 Weeks.

**Additional file 4: Table S2.** WL-A: Summary of the First 5 Weeks.

**Additional file 5: Table S3.** Med-WL: Summary of the First 5 Weeks.

## Data Availability

De-identified results from the clinical laboratory and stool bacterial 16S rRNA amplicon sequencing, targeted qPCR, targeted metabolomics, and exfoliated intestinal cell transcriptomics, as well as information necessary to interpret the data, such as the study protocol and modifications thereof, instruments, survey tools, and variable lists, will be submitted to relevant National Institutes of Health (NIH)-designated data repositories, according to standards set forth in the Health and Human Services Regulations for the Protection of Human Participants and in a manner consistent with the informed consent provided by study participants. Basic clinical metadata such as age will also be provided.

## Abbreviations

ASA24: Automated Self-Administered 24-hour Recall
BA: Bile acids
BMI: Body mass index
CA: Cholic acid
CDCA: Chenodeoxycholic acid
CoD: Coefficient of determination
CRC: Colorectal cancer
DCA: Deoxycholic acid
DE: Differential gene expression
DSMC: Data and safety monitoring committee
EMR: Electronic medical records
ESV: Exact sequence variant
FDR: False discovery rate
GLMM: Generalized linear mixed models
GM-CSF: Granulocyte-macrophage colony-stimulating factor
HbA1c: Hemoglobin A1c
HIPAA: Health Insurance Portability and Accountability Act
HOMA-IR: Homeostatic Model Assessment for Insulin Resistance
hs-CRP: High-sensitivity C-reactive protein
IFN: Interferon
IL: Interleukin
IRB: Institutional Review Board
LCA: Lithocholic acid
LDA: Linear discriminant analysis
Med-A: Mediterranean diet alone
MedDiet: Mediterranean diet
MEPA: Mediterranean Eating Pattern for Americans
NIH: National Institutes of Health
PCR: Polymerase chain reaction
PI: Principal investigator
RCT: Randomized controlled trial
RD: Registered dietitian
sCCA: Sparse Canonical Correlation Analysis
SCFA: Short-chain fatty acid
TDC: Typical diet controls
TNF: Tumor necrosis factor
UI Health: University of Illinois Health and Hospital System.
UIC: University of Illinois Chicago
WL-A: Weight loss alone
