# Supplementary material for: Design of the Building Research in CRC prevention (BRIDGE-CRC) trial: a 6-month, parallel group Mediterranean diet and weight loss randomized controlled lifestyle intervention targeting the bile acid-gut microbiome axis to reduce colorectal cancer risk among African American/Black adults with obesity
Source: Trials. 2023 Feb 15;24:113. doi: 10.1186/s13063-023-07115-4 (PMC9930092; doi:10.1186/s13063-023-07115-4)
Supplement: Supplementary file 2 — Additional file 2. Consent form. [file 13063_2023_7115_MOESM2_ESM.docx]

Leave box empty - For office use only

**University of Illinois at Chicago**

**Mediterranean Diet and Weight Loss: Targeting the Bile Acid/Gut Microbiome Axis to Reduce Colorectal Cancer Risk**

**BRIDGE CRC**

**Principal Investigator/Researcher Name and Title:** Dr. Marian Fitzgibbon

**Department and Institution:** Department of Pediatrics, University of Illinois at Chicago

**Address and Contact Information:** 312-996-0146, mlf@uic.edu

1747 W. Roosevelt Rd. WROB, MC 275, Chicago IL, 60608

**Principal Investigator/Researcher Name and Title:** Dr. Lisa Tussing-Humphreys

**Department and Institution:** Department of Kinesiology and Nutrition, University of Illinois at Chicago

**Address and Contact Information:** 312-355-5521, ltussing@uic.edu

1919 W. Taylor Rd. AHSB, MC 917, Chicago IL, 60612

**Sponsor:** National Institutes of Health/National Cancer Institute

**About this research study**

You are being asked to participate in a research study looking at dietary change and weight loss and how it might impact gut health and colorectal cancer (CRC) risk in overweight African American adults. Research studies answer important questions that might help change or improve the way we do things in the future.

**Taking part in this study is voluntary**

Your participation in this research study is voluntary. You may choose to say “no” to this research or may choose to stop participating in the research at any time. Deciding not to participate, or deciding to stop participating later, will not result in the loss of any services, class standing, and/or professional status to which you are entitled, and will not affect your relationship with the University of Illinois at Chicago (UIC) and/or University of Illinois Hospital and Health Sciences System (UI Health), or any of the agencies or organizations collaborating in this research.

This consent form will give you information about the research study to help you decide whether you want to participate. Please read this form and ask any questions you have before agreeing to be in the study.

You are being asked to be a subject in a research study about how diet and weight affect gut health and CRC risk in overweight/obese African American adults. Specifically, we want to see how 4 different eating pattern and/or weight loss plans affect the bacteria in your gut and your gut health. The 4 different eating and lifestyle plans include:

- A weight loss plan focused on reducing calorie intake and increasing exercise.
- An eating plan focused on adopting the Mediterranean Diet. The Mediterranean Diet encourages people to eat many vegetables and fruits, beans, nuts, whole grains, lean meats and fish, and olive oil as a primary source of fat.
- A weight loss plan that is focused on adopting the Mediterranean Diet and increasing exercise.
- A plan that will not encourage any dietary or physical activity changes.

You have been asked to participate in this research because you are Black or African American, at least 45 years old but not older than 75 years old, are up-to-date with colorectal cancer screening, and you have a body mass index (BMI) of 30 or above. Your participation in this research is voluntary. Your decision whether or not to participate will not affect your current or future dealings with the University of Illinois at Chicago or the University of Illinois Hospital and Health Sciences System (UI Health), or any of the agencies or organizations collaborating in this research. If you decide to participate, you are free to withdraw at any time without affecting that relationship. Approximately 250 subjects may be involved in this research at UIC.

**Important Information**

This information gives you an overview of the research. More information about these topics may be found in the pages that follow.

| **WHY IS THIS STUDY BEING DONE?** | You are being asked to participate in a research study looking at dietary change and weight loss and how it might impact gut health and colorectal cancer (CRC) risk in overweight Black or African American adults. |
| --- | --- |
| **WHAT WILL I BE ASKED TO DO DURING THE STUDY?** | If you are eligible and agree to be a part of this study, you will be randomly assigned to one of four groups. Three of the groups will include classes. The fourth group does not require you to attend classes, but instead will set up one meeting with a nutritionist.  You will be randomly assigned into one of four groups:  **Weight Loss:** This group will focus on lifestyle changes that will encourage eating fewer calories and exercising regularly to lose weight. This group will have approximately 20 people, and will meet weekly with a nutrition professional either on the phone or in person for 6 months (24 sessions). This group will also include an effort to increase exercise  **Med-Diet:** This group will focus on adopting a Mediterranean Diet, a largely plant-based diet, focused on eating many vegetables and fruits, beans, nuts, whole grains, lean meats and fish, and olive oil as a primary source of fat. This group will have approximately 20 people, and will meet weekly with a nutrition professional either on the phone or in person for 6 months (24 sessions). You will be asked to maintain your current level of physical activity.  **Med-Diet + Weight Loss**: This group is similar to the Med-Diet group, but with additional focus on eating fewer calories and exercising to lose weight. This group will have approximately 20 people, and will meet weekly with a nutrition professional either on the phone or in person for 6 months (24 sessions). This group will also include an effort to increase exercise..  **Traditional Diet**: People randomly selected for this group will meet with a nutrition professional either on the phone or in person for 60 minutes who will help participants to maintain current eating and activity patterns and weight over the next 6 months. This group will receive weekly health newsletters, but no dietary or physical activity recommendations will be provided during the study. This group will receive all the materials of the Med Diet + Weight Loss group following completion of the study.  **Data Collection**  In order to participate in the research, you will need to come to our offices at the University of Illinois to complete data collection. First, we will need to complete a screening to make sure you are eligible for the research study. This should take about 45 minutes. We will measure your height and weight, and ask you questions about your health.  If you are eligible and interested, we will immediately begin the data collection process. We will ask you about your diet, provide you with a home stool collection kit, provide you with a physical activity monitor to wear for one week, and schedule your next in-person visit. Before your next visit, we will send you surveys to complete online where we will ask you questions about your health and lifestyle, including how you usually eat, and how you feel. Those questions will take between 60 and 90 minutes to complete. During the next visit, we receive your stool sample, draw some blood, measure your blood pressure, complete diet related surveys and do a DXA body composition scan. Your total visit will be approximately 2-3 hours.  We will ask you to repeat this data collection 2 more times over the next 6-8 months. We will conduct data collection at the beginning of the study, at the mid-point of the intervention (3 months) and when the class is complete (6 months).  For more information, please see the “What Procedures Are Involved?” section below. |
| **HOW MUCH TIME WILL I SPEND ON THE STUDY?** | Each data collection period will require at least 2 visits to our offices at UIC. The screening/data collection visit is expected to take about 1-1.5 hours. Each of the 3 main data collection visits will take about 3 hours. Altogether, the data collection visits will take about 10 hours to complete.  If you are randomized to one of the groups that meets approximately every week for a diet/lifestyle class, you will be asked to meet for 24 sessions at a community location or virtually online. Each class will be either 60 or 90 minutes in length. |
| **ARE THERE ANY BENEFITS TO TAKING PART IN THE STUDY?** | You may or you may not directly benefit from participation in this study. Researchers may learn new things that will help others. |
| **WHAT ARE THE MAIN RISKS OF THE STUDY?** | There are some potential risks associated with participating in this research:   1. Discomfort or bruising during/after the blood draw 2. Feeling hungry while fasting for 12 hours before the blood draw. You may also feel some stomach discomfort as you change your diet as part of the research study. 3. Temporary fatigue, soreness or injuries like muscle strains or sprains, and even cardiovascular events, including lightheadedness to heart attack from exercise class. 4. There is risk of some radiation exposure from the DXA scan, the equivalent to the radiation you would receive taking a 6-hour airplane flight.   For details and a list of risks you should know about, please see the “What Are the Potential Risks and Discomforts of the Study” section below. |
| **DO I HAVE OTHER OPTIONS BESIDES TAKING PART IN THE STUDY?** | This is not a treatment study. Your alternative is not to participate in this study. Additionally, exercise and nutritional guidance are commercially available outside of participation in this study. |
| **QUESTIONS ABOUT THE STUDY?** | Contact the researcher Marian Fitzgibbon at (312) 996-9028 or email address mlf@uic.edu   - if you have any questions about this study or your part in it, - if you feel you have had a research-related injury, - if you have questions, concerns or complaints about the research. |

**Please review the rest of this document for details about these topics and additional things you should know before making a decision about whether to participate in this research. Please also feel free to ask the researchers questions at any time.**

**What procedures are involved?**

This research will be performed at our offices at the University of Illinois at Chicago, located at 1747 W. Roosevelt Road and 1640 W. Roosevelt Road, Chicago, IL 60608. The classes will take place in a community location to be determined.

If you are eligible and agree to be a part of this study, you will be randomly assigned to one of four groups. Three of the groups will include classes that will be offered both virtually and at a community location (to be determined). The fourth group does not require you to attend classes, but instead you will meet one on one with a registered dietitian on one occasion.

You will be randomly assigned into one of four groups:

**Weight Loss:** This group will focus on lifestyle changes that will encourage eating fewer calories and exercising regularly to lose weight. This group will have approximately 20 people, and will meet weekly with a nutrition professional either on the phone or in person for 6 months (24 sessions). This group will also include an effort to increase exercise.

**Med-Diet:** This group will focus on adopting a Mediterranean Diet, a largely plant-based diet, focused on eating many vegetables and fruits, beans, nuts, whole grains, lean meats and fish, and olive oil as a primary source of fat. This group will have approximately 20 people, and will meet weekly with a nutrition professional either on the phone or in person for 6 months (24 sessions). You will be asked to maintain your current level of physical activity.

**Med-Diet + Weight Loss**: This group is similar to the Med-Diet group, but with additional focus on eating fewer calories and exercising to lose weight. This group will have approximately 20 people, and will meet weekly with a nutrition professional either on the phone or in person for 6 months (24 sessions). This group will also include an effort to increase exercise.

**Traditional Diet**: People randomly selected for this group will meet with a nutrition professional for 60 minutes either on the phone or in person. The nutrition professional will help participants to maintain current eating and activity patterns and weight over the next 6 months. This group will receive weekly health newsletters, but no dietary or physical activity recommendations will be provided during the study. This group will receive all the materials of the Med Diet + Weight Loss group following completion of the study.

We may want to video record some classes for future training purposes. This is optional and you can refuse to be recorded if you prefer not to be.

**Data Collection**

**Screening**: In order to participate in the research, you will need to come to our offices at the University of Illinois Chicago. First, we will need to complete some screening to make sure you are eligible for the research study. This should take about 45 minutes. We will measure your height and weight and ask you questions about your health. We may also ask you to show proof (from your doctor or medical record patient portal) that you are up to date with colorectal cancer screening.

During screening we will also ask you if you have had a Covid-19 infection within the past 6 weeks. If you have, you will need to wait 6 weeks from diagnosis before you can complete data collection.

**Screening involves:** Conducting informed consent, measurement of height and weight, questionnaires.

**Full-study data collection**: If you are eligible and interested, we will immediately begin the data collection process. We will ask you questions about your diet.give you a stool sample collection kit with instructions, and ask you wear an activity monitor.. Before your next data collection visit, we will send you some surveys with questions to complete online. We will ask you questions about your health and lifestyle, including how you usually eat, and how you feel. Those questions will take 1-1.5 hours to complete. At the second visit, you will return your stool sample and we will draw some blood, measure your blood pressure, ask you about your diet, do a DXA body composition scan.Your total visit will be approximately 2-3 hours. When we draw your blood, one of the measures we will be looking at is A1C (a measure of blood sugar). If your A1C is found to be above 9, you will be ineligible for the study, and we will call to notify you in this event.

We will ask you to repeat this data collection 2 more times over the next 6-months. We will conduct data collection at the beginning of the study, at the mid-point of the intervention (3 months) and when the class is complete (6 months).

**Data collection involves:**

1. **Venous blood collection**: A person who is trained and experienced in drawing blood will complete your blood collection. We will need you to fast for at least 12 hours and avoid various prescribed and over the counter medications. (10 minutes) We will draw approximately 4 tablespoons of blood.
2. **Stool collection:** We will provide you with a stool collection kit and instructions for you to collect a stool sample at home.
3. **Measurements**: We will measure your blood pressure, height, and weight. (10 minutes)
4. **Questionnaires**: You will complete a series of interviewer-administered questionnaires including questions relating to demographics, behavior, diet, psychosocial measures, and cognitive variables. (2.5 hours)
5. **Body composition:** We will collect body composition data with a DXA scan. We will be using the Lunar iDXA (GE Healthcare), a state-of-the-art scanner that provides precise, non-invasive measures of how your body is composed. From the scan, we will see how much of your body is made up of bone, muscle and fat mass. A whole body DXA scan takes about 10 minutes, during which time you will be asked to lie still on an exam table. This scan will take place at the Integrative Physiology Lab at the University of Illinois at Chicago. The amount of radiation exposure is less than the dose received during a trans-Atlantic flight.
6. **Activity monitors**: We will ask you to wear a Fit Bit (a small device that measures movement) for 7 days so that we can measure your physical activity, sleep and heart rate. It is a small device that you wear on your wrist, like a watch. We will collect data remotely using an application called Fit-a-base, that will not be able to see your activity information but will allow us to see it only during the period we tell you we are collecting data. The FitBit will be yours to keep after we have collected data for this study. You will be able to see your own activity information when we connect the Fit Bit to your phone and/or email.

**Does the study involve genetic testing?** In this study, we are looking at how genes in your colon respond to the interventions (diet and/or weight loss). However, we are not conducting genetic testing to understand your risk for certain diseases. For your knowledge, the cells of your body contain deoxyribonucleic acid or DNA for short. DNA is passed down from your parents. It carries the genes that determine physical features such as the color of your hair and eyes. Differences in our genes help explain why we all look different. They may also determine how different people get certain diseases and respond to treatments including diet and weight loss. In this study, we will look at how genes in your colon function before and after the interventions. We will compare data we collect at 3 months and after the interventions to what we observed at the start of the study. The genetic data will be obtained from tissue from your intestine found in your stool sample.

In accordance with our study sponsor the National Institutes of Health (NIH), we will submit your deidentified gene expression data from your colon to a public repository approved by NIH. NIH is a national research agency and is part of the federal government.

A repository contains information from many people. Other researchers can take information from the repository and use it in their studies. Their studies may be like this one or may be completely different. We will provide basic information such as sex, race/ethnicity, and age so that the data can be compared to colon genomic data from other studies. Because the data will be de-identified, it can no longer be linked back to you and therefore cannot be withdrawn from further use.

We will label your genomic data with a code, instead of your name or other information that people could use to directly identify you. Even so, there is a possibility that when your genomic information is combined with other information available to researchers, either now or in the future, they may be able to identify a group you belong to (like an ethnic group or a disease population) or, less likely, you personally. NIH prohibits people from trying to identify individuals whose genomic information is in an NIH-designated repository.

Researchers will have *unrestricted access* to your specific genomic information. Unrestricted access means that researchers may obtain genomic information from the repository without special approval from NIH.

**Will I receive any psychological, health, and/or biospecimen test results from the study?**

We can share some data if requested, such as BMI, cholesterol and/or A1C and the DXA body scan. We cannot share specific results of some other tests, though we will inform you if any testing indicates the need for follow-up with your doctor.

**What are the potential risks and discomforts of the study?**

There are some potential risks associated with participating in this research:

1. **Discomfort during blood draw**: Some participants may be uncomfortable with having their blood drawn and may feel lightheaded or dizzy or even faint. Other risks and discomforts from a blood draw include pain or bruising at the site of the stick and risks of infection at this site.
2. **Discomfort or embarrassment with handling stool sample collection.** We will provide a kit with instructions and tools to mitigate discomfort.
3. **Fasting and refraining from medications:** You will be asked to fast and refrain from taking any anti-inflammatory, COX-2 inhibitors, steroids, and medication for blood sugar or cholesterol for 12 hours prior to blood draw. Resulting side effects may include increased pain, headache, tiredness, weakness, upset stomach, anxiety, and hunger.
4. **Distress when answering questions**: responding to study questionnaires is not expected to but may provoke uncomfortable feelings or cause mild distress. If you experience any of these symptoms, you may choose not to answer any question that may cause you discomfort, or you may choose to withdraw from the study.
5. **Loss of confidentiality**: This study includes collection of identifiable health information and as with any study, there is the risk of a breach of confidentiality.
6. **Injury risk:** Participation in the exercise component could place you at increased risk for exercise-related injuries. Treatment may be obtained through: UIC Medical Center, your regular doctor or the treatment center or clinic of your choice. Costs associated with that treatment will not be paid by the research project.
7. **Muscle fatigue**: There is a risk of temporary soreness and fatigue from increasing your exercise if you are randomized into a group that includes exercise.
8. **GI distress:** There is a risk of gastrointestinal distress or hunger pains from the changes in diet associated with this study.
9. **DXA scan:** There is risk of some radiation exposure from the DXA scan. The amount of radiation you might receive from one scan is equivalent to the amount you would receive taking a trans-Atlantic airplane flight. If you are pregnant, you will not receive a DXA scan. We will provide a pregnancy test for you if you have menstruated in the past 6 months.
10. **Imaging:** If you agree to be photographed or recorded with video, there is an increased risk of loss of privacy and confidentiality.
11. **Unknown risk:** While we believe that the risks to you and your family are very low, we are not able to know all of the risks from taking part in genetic research studies. Your privacy will be protected to the fullest extent possible. In addition, there may be undue stress, anxiety, or embarrassment resulting from inadvertent disclosure of information on family relationships, ethnic heritage, or potentially stigmatizing conditions.

**Will I be told about new information that may affect my decision to participate?**

During the course of the study, you will be informed of any significant new research information (either good or bad), such as changes in the risks or benefits resulting from participation in the research or new alternatives to participation, that might cause you to change your mind about continuing in the research. If new information is provided to you, your consent to continue participating in this research may be re-obtained.

**What if I am injured as a result of my participation?**

If you get ill or injured from being in the study, UIC will help you get medical treatment. You should let the study doctor know right away that you are ill or injured. If you believe you have become ill or injured from this study, you should contact Dr. Marian Fitzgibbon at (312) 996-0146.

You should let any health care provider who treats you know that you are in a research study. If you do seek medical treatment, please take a copy of this document with you because it may help the doctors where you seek treatment to treat you. It will also provide the doctors where you seek treatment with information they may need if they want to contact the research doctors.

You or your health insurance plan will be billed. No money has been set aside to pay the costs of this treatment. Health insurance plans may or may not cover costs of research-related injury or illness. You should check with your insurance company before deciding to participate in this research study. Costs not covered by insurance could be substantial.

UIC has not set aside any money to pay you or to pay for your treatment if you get ill or injured from being in the study. There are no plans for the University to provide other forms of compensation (such as lost wages or pain and suffering) to you for research related illnesses or injuries. The only exception to this policy is if it is proven that your injury or illness is directly caused by the negligence of UIC.

By signing this form, you are not giving up any legal rights to seek compensation of injury.

**What about privacy and confidentiality?**

Efforts will be made to keep your personal information confidential; however, we cannot guarantee absolute confidentiality. In general, information about you, or provided by you, during the research study, will not be disclosed to others without your written permission. However, laws and state university rules might require us to tell certain people about you. For example, study information which identifies you and the consent form signed by you may be looked at and/or copied for quality assurance and data analysis by:

- Representatives of the university committee and office that reviews and approves. research studies, the Institutional Review Board (IRB) and Office for the [Protection of Research Subject](http://illinois.edu/ds/detail?departmentId=illinois.eduNE344&search_type=all&skinId=0&sub=)s.
- Other representatives of the State and University responsible for ethical, regulatory, or financial oversight of research.
- Government Regulatory Agencies, such as the Office for Human Research Protections (OHRP).
- The National Institutes of Health.

A possible risk of the research is that your participation in the research or information about you might become known to individuals outside the research. To keep data secure, all datasets will be stored on a password-protected server, and any paper records will be stored in locked cabinets in the research office. Your individual data will be stripped of all direct and indirect identifiers or destroyed after analysis.

When the results of the research are published or discussed in conferences, no information will be included that would reveal your identity.

Please remember that there is an exception to protecting subject privacy and confidentiality if child, elder, and/or disabled adult abuse or neglect of an identifiable individual, or the threat of imminent self-harm or harm to others is disclosed. If such information is disclosed, the researchers may be obligated to inform the appropriate authorities.

Confidentiality cannot be guaranteed if you indicate that you intend to commit future criminal conduct, you intend to harm yourself or someone else, or if you are an inmate and intend to leave the facility without authorization.A description of this study will be available on [http://www.ClinicalTrials.gov](http://www.clinicaltrials.gov/), as required by U.S. law.  This website will not include information that can identify you.  At most, the website will include a summary of the results.  You can search this website at any time.

There is a Federal law called the Genetic Information Nondiscrimination Act (GINA). In general, this law makes it illegal for health insurance companies, group health plans, and most employers to discriminate against you based on your genetic information. However, it does not protect you against discrimination by companies that sell life insurance, disability insurance, or long-term care insurance. For example, life insurance companies may charge a higher rate based on this information. GINA also does not protect you against discrimination if you have already been diagnosed with the genetic disease that is being tested in this research study.

**What are the costs for participating in this research?**

There are no costs to you for participating in this research.

**Will I be reimbursed for any of my expenses or paid for my participation in this research?**

If your pre-screening results do not make you eligible to safely participate in this study, we will reimburse you $10 for your time.

If you complete the interview and data collection, you will be reimbursed $70 for your full participation in the first data collection period, $70 for your participation in the second data collection period and $100 for your participation in the third data collection period. Therefore, you may be reimbursed a total of $240 over the course of 6 months if you complete all phases of the research. If you do not finish the study, you will be compensated for the visits you have completed. You will be paid in cash at the end of each visit.

If you are randomly assigned to group that includes 24 weekly meetings and online video and activities , you will have the opportunity to receive home delivered meal recipe boxes and other health lifestyle incentives worth a total of $240.. If you are in the group that will not ask you to change your lifestyle, the Traditional Diet group, you will receive equivalent value in cash, $240 over the course of the 6 month intervention. You will receive this payment within approximately 30 days of the end of the study by mail or in person. We may need to collect your social security number or Taxpayer Identification Number (TIN) in order to issue your compensation and for tax reporting purposes to the United States Internal Revenue Service (IRS).

**Can I withdraw or be removed from the study?**

If you decide to participate, you have the right to withdraw your consent and leave the study at any time without penalty.

The researchers and/or funder also have the right to stop your participation in this study without your consent if:

- They believe it is in your best interests;
- You were to object to any future changes that may be made in the study plan.

If you choose to no longer be in the study and you do not want any of your future information to be used, you must inform the researcher Dr. Marian Fitzgibbon in writing at the address on the first page. The researcher Dr. Marian Fitzgibbon may still use your information that was collected prior to your written notice.

**Remember**:

Your participation in this research is voluntary. Your decision whether or not to participate will not affect your current or future relations with the University. If you decide to participate, you are free to withdraw at any time without affecting that relationship.

**Signature of Subject**

I have read (or someone has read to me) the above information. I have been given an opportunity to ask questions and my questions have been answered to my satisfaction. I agree to participate in this research. I will be given a copy of this signed and dated form.

Signature Date

Printed Name

Signature of Person Obtaining Consent Date (must be same as subject’s)

Printed Name of Person Obtaining Consent

I have read the above information. I have been given an opportunity to contact the researchers and ask questions, and my questions have been answered to my satisfaction. I agree to participate in this research. **PLEASE PRINT OUT A COPY OF THIS DOCUMENT FOR YOUR RECORDS.**

**Future Research**

As newer laboratory technology emerges, researchers might want to perform additional tests on any remaining blood and stool samples that you provided for this study, which will be stored long term if you agree (a minimum of 6 years after the study has concluded). This could help us learn more about colorectal health and the microbiome. If you agree, your samples will be stored with linked codes in a secure password protected database owned by the Principal Investigator and the study staff. Any future use of the samples would be reviewed and approved by an Institutional Review Board.

I agree to allow samples of my blood and stool to be used for future research including genomic research.

Initials_______

I do not agree to allow samples of my blood and stool to be used for future research*.*

*Initials_______*

**Samples would be kept by the research team at *Dr. Lisa Tussing’s lab at the University of Illinois at Chicago, College of Applied Health Sciences, 1919 W. Taylor Street, Lab 106, Chicago IL, 60612*, for future use in research.**

We often have research studies looking for participants meeting your eligibility criteria. Following your participation with this research study, do you wish to be contacted about future research? As always, your future participation in any research study would be completely voluntary and would not affect your medical care.

I agree to the possibility of being contacted for future research studies.

*Initials ________*

I DO NOT wish to be contacted for future research studies.

*Initials ________*

**Photographs and Video**

Do you agree to allow the use of photographs/video recording to be taken during the course of the Bridge CRC Trial for training and publication purposes, (including publications, presentation or broadcast via newspaper, internet or other media sources)? If you consent it is with full knowledge that you waive all claims for compensation for use, or for damages.

I agree to the possibility of being photographed or video recorded over the course of the BRIDGE CRC Study.

*Initials ________*

I DO NOT agree to the possibility of being photographed or video recorded over the course of the BRIDGE CRC study.

*Initials ________*
