# Supplementary material for: Design of the Building Research in CRC prevention (BRIDGE-CRC) trial: a 6-month, parallel group Mediterranean diet and weight loss randomized controlled lifestyle intervention targeting the bile acid-gut microbiome axis to reduce colorectal cancer risk among African American/Black adults with obesity
Source: Trials. 2023 Feb 15;24:113. doi: 10.1186/s13063-023-07115-4 (PMC9930092; doi:10.1186/s13063-023-07115-4)
Supplement: Supplementary file 3 — Additional file 3: Table S1. Med-A: Summary of the First 5 Weeks. [file 13063_2023_7115_MOESM3_ESM.docx]

| **Supplementary Table 1**  **Med-A: Summary of the First 5 Weeks** | | | | | |
| --- | --- | --- | --- | --- | --- |
| **Med-A** | **Individual Session**  **(In-person, Zoom, Phone)** | **Individual Session length (min)** | **Asynchronous Content**  **(Facebook Private page or emailed to participant)** | **Challenge** | **Food Delivery** |
| Session 1 | Objective:  Introduce goals of Bridge-CRC trial  Introduce specifics of intervention group  Introduce how to use intervention tools  Handouts:  MedDiet exchange list  MedDiet exchange booklet  MedDiet tracking log | 60 | Videos:  Knife skills  How to count exchanges using a sample meal  Infographic:  MedDiet and CRC risk | No | No |
| Session 2 | Objective:  Discuss associations between diet and CRC  Review MedDiet exchange list and examples  Review self-monitoring reporting  Discuss diet successes and challenges over the past week | 30 | Videos:  How to count exchanges for balsamic glazed vegetables  How to cook balsamic glazed vegetables  Infographic:  5 tips on how to roast any vegetable | Cook and post or send a picture of balsamic glazed vegetables | Yes |
| Session 3 | Objective:  Discuss the successes and challenges to meeting your MedDiet exchange goals and using the MedDiet exchange list  Review the MedDiet exchange booklet  Handouts:  MedDiet exchange booklet | 30 | Videos:  How to make an olive oil vinaigrette  The MedDiet minute  Infographics:  The 9 MedDiet groups  Olive oil | Prepare and post or send a picture of your homemade olive oil vinaigrette | No |
| Session 4 | Objective:  Discuss diet successes and challenges over the past week.  Overview of what to eat on the MedDiet  Discuss MedDiet meal planning using the weekly meal planning worksheet and MedDiet shopping list  Discuss MedDiet grocery shopping guide  Handouts:  Meal planning worksheet  MedDiet shopping guide | 30 | Videos:  How to count exchanges for the tuna and grain salad  How to make a tuna and grain salad  Infographics:  In season buying guide  MedDiet food swaps | Cook and post or send a picture of tuna and grain salad | Yes |
| Session 5 | Objective:  Discuss diet successes and challenges over the past week  Discuss successes and challenges to adopting a MedDiet  Review MedDiet exchange list  Review MedDiet basics | 30 | Videos:  MedDiet minute  How to cook lean proteins  Infographic:  Tips to selecting MedDiet proteins | Cook and post or send a picture of lean protein | No |
