# Supplementary material for: Design of the Building Research in CRC prevention (BRIDGE-CRC) trial: a 6-month, parallel group Mediterranean diet and weight loss randomized controlled lifestyle intervention targeting the bile acid-gut microbiome axis to reduce colorectal cancer risk among African American/Black adults with obesity
Source: Trials. 2023 Feb 15;24:113. doi: 10.1186/s13063-023-07115-4 (PMC9930092; doi:10.1186/s13063-023-07115-4)
Supplement: Supplementary file 4 — Additional file 4: Table S2. WL-A: Summary of the First 5 Weeks. [file 13063_2023_7115_MOESM4_ESM.docx]

| **Supplementary Table 2**  **WL-A: Summary of the First 5 Weeks** | | | | | | |
| --- | --- | --- | --- | --- | --- | --- |
| **WL-A** | **Individual Session**  **(In-person, Zoom, Phone)** | **Individual Session length (min)** | **Asynchronous Content**  **(Facebook Private page or emailed to participant)** | **Physical Activity**  **Steps/FitBit** | **Challenge** | **Food Delivery** |
| Session 1 | Objective:  Introduce goals of Bridge-CRC trial  Introduce specifics of intervention group  Introduce how to use intervention tools  Introduce how to track and report food and physical activity  Handouts:  How to add a friend on FitBit | 60 | Video:  Step-by-Step Using the Cronometer App video  Infographics:  Connecting body weight to CRC  How to add a friend on FitBit | Review FitBit and introduce step goal | No | No |
| Session 2 | Objective:  Discuss diet and physical activity successes and challenges over the past week  Discuss portion control  Discuss 3 key strategies to achieving weight loss through food  The importance of self-monitoring for weight loss success  Handouts:  Portion control | 30 | Videos:  How to measure foods  How drastically food portions have changed in the past 20 years | Introduction to fitness instructor | Oven frying challenge | No |
| Session 3 | Objectives:  Discuss diet and physical activity successes and challenges over the past week  Be a calorie detective: Reading the nutrition facts label  Discuss how and why to measure foods  Handout:  Read the nutrition facts label | 30 | Video:  Reading the nutrition facts label  Infographic:  Increasing your steps | Motivational video from fitness instructor | Increase 500 steps per day | No |
| Session 4 | Objectives:  Discuss diet and physical activity successes and challenges over the past week  Eating fewer calories tips and tricks  Tips for increasing daily physical activity  Discuss mindful eating  Handout:  Healthy eating | 30 | Videos:  Mindful eating  Healthy fats on a food label  Infographic:  Reasons to engage in PA | Counseling on meeting step goals | Increase 500 steps per day | No |
| Session 5 | Objectives:  Discuss diet and physical activity successes and challenges over the past week  Discuss reducing intake and staying on track with weight loss goals  Discuss the use of the WL-A tools  Build a better recipe  Handouts:  Making healthier food choices  Build a better recipe | 30 | Video:  10 healthy food swaps  Infographic:  List of physical activities | Motivational video from fitness instructor | Increase 500 steps per day | No |
