# Supplementary material for: Design of the Building Research in CRC prevention (BRIDGE-CRC) trial: a 6-month, parallel group Mediterranean diet and weight loss randomized controlled lifestyle intervention targeting the bile acid-gut microbiome axis to reduce colorectal cancer risk among African American/Black adults with obesity
Source: Trials. 2023 Feb 15;24:113. doi: 10.1186/s13063-023-07115-4 (PMC9930092; doi:10.1186/s13063-023-07115-4)
Supplement: Supplementary file 5 — Additional file 5: Table S3. Med-WL: Summary of the First 5 Weeks. [file 13063_2023_7115_MOESM5_ESM.docx]

| **Supplementary Table 3**  **Med-WL: Summary of the First 5 Weeks** | | | | | | |
| --- | --- | --- | --- | --- | --- | --- |
| **Med-WL** | **Individual Session**  **(In-person, Zoom, Phone)** | **Individual Session length (min)** | **Asynchronous Content**  **(Facebook Private page or emailed to participant)** | **Physical Activity**  **Steps/Fitbit** | **Challenge** | **Food Delivery** |
| Session 1 | Objective:  Introduce goals of Bridge-CRC trial  Introduce specifics of intervention group  Introduce how to use intervention tools  Handouts:  MedDiet exchange list  MedDiet exchange booklet  MedDiet tracking log | 60 | Videos:  Knife skills  How to count exchanges using a sample meal  Infographics: Description of a MedDiet  Body weight and CRC risk | Review FitBit and introduce step goal | No | No |
| Session 2 | Objective:  Discuss associations between body weight and CRC  Discuss portion control  Review MedDiet exchange list and examples  Review self-monitoring reporting  Discuss diet and physical activity successes and challenges over the past week | 30 | Videos:  How to cook balsamic glazed vegetables  How to count exchanges for balsamic glazed vegetables  Infographics:  How to increase steps  MedDiet and CRC risk | Introduction to fitness instructor | Cook and post or send a picture of balsamic glazed vegetables | Yes |
| Session 3 | Objective:  Discuss the successes and challenges to meeting your MedDiet exchange goals and using the MedDiet exchange list and physical activity goals  Review the MedDiet exchange booklet  Handouts:  MedDiet exchange booklet | 30 | Video:  How to make an olive oil vinaigrette  Infographics:  Types of seasoning (salt, fat, acid, sweet, heat)  Reasons to engage in PA | Motivational video from fitness instructor | Increase steps by 500 per day | No |
| Session 4 | Objective:  Discuss diet and physical activity successes and challenges over the past week.  Overview of what to eat on the MedDiet  Discuss MedDiet meal planning using the Weekly meal planning worksheet  Introduce mindfulness for diet intake  Discuss strategies to increase physical activity  Handouts:  Meal planning worksheet  MedDiet shopping guide | 30 | Videos:  How to make a tuna and grain salad  How to count exchanges for the tuna and grain salad  Infographics: Mindful eating  MedDiet food swaps | Counseling on meeting step goals | Cook and post or send a picture of tuna and grain salad | Yes |
| Session 5 | Objective:  Discuss diet and physical activity successes and challenges over the past week  Discuss reducing intake and staying on track with weight loss goals  Discuss the use of the Med-WL tools  Provide counseling on ways to increase physical activity | 30 | Video:  How to cook lean proteins  Infographics:  Food safety tips  Additional physical activity resources | Motivational video from fitness instructor | Increase steps by 500 per day | No |
